# Impaired SNF2L Chromatin Remodeling Prolongs Accessibility at Promoters Enriched for Fos/Jun Binding Sites and Delays Granule Neuron Differentiation

**DOI:** 10.3389/fnmol.2021.680280

**Published:** 2021-07-06

**Authors:** Laura R. Goodwin, Gerardo Zapata, Sara Timpano, Jacob Marenger, David J. Picketts

**Affiliations:** ^1^Regenerative Medicine Program, Ottawa Hospital Research Institute, Ottawa, ON, Canada; ^2^Department of Biochemistry, Microbiology and Immunology, University of Ottawa, Ottawa, ON, Canada; ^3^Cellular and Molecular Medicine, University of Ottawa, Ottawa, ON, Canada

**Keywords:** SMARCA1, Snf2L, ISWI chromatin remodeler, cerebellar granule neuron progenitors, ATAC-seq, chromatin accessibility

## Abstract

Chromatin remodeling proteins utilize the energy from ATP hydrolysis to mobilize nucleosomes often creating accessibility for transcription factors within gene regulatory elements. Aberrant chromatin remodeling has diverse effects on neuroprogenitor homeostasis altering progenitor competence, proliferation, survival, or cell fate. Previous work has shown that inactivation of the *ISWI* genes, *Smarca5* (encoding Snf2h) and *Smarca1* (encoding Snf2l) have dramatic effects on brain development. *Smarca5* conditional knockout mice have reduced progenitor expansion and severe forebrain hypoplasia, with a similar effect on the postnatal growth of the cerebellum. In contrast, *Smarca1* mutants exhibited enlarged forebrains with delayed progenitor differentiation and increased neuronal output. Here, we utilized cerebellar granule neuron precursor (GNP) cultures from *Smarca1* mutant mice (Ex6DEL) to explore the requirement for Snf2l on progenitor homeostasis. The Ex6DEL GNPs showed delayed differentiation upon plating that was not attributed to changes in the *Sonic Hedgehog* pathway but was associated with overexpression of numerous positive effectors of proliferation, including targets of Wnt activation. Transcriptome analysis identified increased expression of *Fosb* and *Fosl2* while ATACseq experiments identified a large increase in chromatin accessibility at promoters many enriched for Fos/Jun binding sites. Nonetheless, the elevated proliferation index was transient and the Ex6DEL cultures initiated differentiation with a high concordance in gene expression changes to the wild type cultures. Genes specific to Ex6DEL differentiation were associated with an increased activation of the ERK signaling pathway. Taken together, this data provides the first indication of how *Smarca1* mutations alter progenitor cell homeostasis and contribute to changes in brain size.

## Introduction

Normal brain development requires the neural progenitors to interpret external cues that are used to remodel their chromatin and modulate an intrinsic gene expression program that ultimately defines cellular competence, regulates proliferation, and/or initiates a cell fate lineage decision. Indeed, progenitor cell homeostasis is a tightly regulated process that if perturbed can affect brain size, cellular fate and lamination, and/or cause neurodevelopmental disorders. The characterization of animal models have shown that ablation of chromatin remodeling factors significantly impact neurogenesis, while human genetic studies have implicated them in numerous neurodevelopmental disorders (Bogershausen and Wollnik, 2018; Goodwin and Picketts, 2018; Sokpor et al., 2018; Timpano and Picketts, 2020).

Chromatin remodeling is catalyzed by conserved complexes containing a subunit with a SNF2-like helicase domain that binds and hydrolyzes ATP to reposition nucleosomes. These complexes can be subdivided into four classes, the switch/sucrose non-fermenting (SWI/SNF), imitation-switch (ISWI), chromodomain helicase DNA binding (CHD), and inositol requiring 80-like (INO80) families based on extended homology within the ATPase domain and the inclusion of additional motifs that facilitate chromatin interactions (Clapier et al., 2017). The mammalian ISWI family comprises the closely related *SMARCA1* and *SMARCA5* genes that are orthologs of the *Drosophila ISWI* gene and encode the SNF2L and SNF2H proteins, respectively. The mammalian ISWI proteins form heterodimers comprised of either SNF2H or SNF2L and, most often, a *BAZ* (bromodomain adjacent to zinc finger) gene family member (Goodwin and Picketts, 2018). Seven complexes have been purified including the NURF (Nucleosome remodeling factor), ACF (ATP-utilizing chromatin assembly factor) and CHRAC (chromatin assembly complex) complexes that are highly conserved across species (Tsukiyama and Wu, 1995; Ito et al., 1997; Varga-Weisz et al., 1997). In addition, four novel complexes have been purified from mammalian cells, namely the WICH (WSTF-ISWI chromatin remodeling complex), CERF (CECR2-containing remodeling factor), RSF (remodeling and spacing factor), and NoRC (Nucleolar remodeling complex) complexes (LeRoy et al., 1998; Strohner et al., 2001; Bozhenok et al., 2002; Banting et al., 2005). While it was originally reported that five complexes contained SNF2H (ACF, CHRAC, NoRC, RSF, WICH) and two comprised SNF2L (CERF, NURF), a recent study has suggested that SNF2H and SNF2L are interchangeable within all seven complexes and identified a novel eighth complex containing the protein BAZ2A (Oppikofer et al., 2017). However, most genetic and biochemical data indicate that *Smarca1* and *Smarca5* have non-redundant functions, as described below.

The ISWI proteins remodel and space nucleosome arrays *in vitro* albeit with different affinities (Corona et al., 1999; Langst et al., 1999; Tang et al., 2004; Leonard and Narlikar, 2015; Clapier et al., 2017), and have different effects on the positioning of nucleosomes at transcriptional start sites (TSS) and at CTCF and other transcription factor binding sites (Qiu et al., 2015; Kwon et al., 2016; Wiechens et al., 2016). Moreover, SNF2H containing complexes facilitate DNA repair, regulate heterochromatin maintenance and coordinate rRNA gene expression; functions not demonstrated for SNF2L complexes (as reviewed by Goodwin and Picketts, 2018). However, distinct roles seem less clear when examining mouse models. The inactivation of *Bptf*, the gene encoding the largest subunit of the Snf2l-containing NURF complex results in decreased progenitor self-renewal and impaired terminal differentiation in multiple cell types (Landry et al., 2008, 2011; Koludrovic et al., 2015; Frey et al., 2017). Similarly, inactivation of the *Smarca5* gene resulted in reduced growth and pre-implantation lethality, while tissue specific-inactivation in the developing brain resulted in mice with a striking cerebellar hypoplasia caused by a drastically reduced proportion of proliferating cells in the external granule cell layer (Stopka and Skoultchi, 2003; Alvarez-Saavedra et al., 2014). Given that Snf2l is also expressed in the granule neurons of the cerebellum, it provides the opportunity to utilize granule neuron progenitor cultures to define whether Snf2h and Snf2l act with overlapping or separate functions in progenitor cell homeostasis.

The development of the cerebellum is dependent on two distinct progenitor populations, one located in the ventricular zone (VZ) lining the fourth ventricle and one in the upper rhombic lip (Hatten and Heintz, 1995). The VZ progenitors give rise to all the inhibitory neurons, including Purkinje neurons, while some of these Nestin-expressing progenitors migrate to the external granule layer (EGL) to produce Bergmann glia (Hatten and Heintz, 1995; Hoshino et al., 2005). The progenitors from the rhombic lip give rise to all excitatory neurons including those that form the deep cerebellar nuclei and the population of granule neuron precursors (GNPs) that migrate to the EGL where they proliferate extensively in the postnatal period to produce granule neurons and promote folia growth (Hatten and Heintz, 1995; Alder et al., 1996; Machold and Fishell, 2005). *Smarca5* is expressed robustly in the rhombic lip and the VZ in the embryo but it only seems to affect GNP expansion as normal numbers of Purkinje neurons were detected in the hypoplastic cerebellum of *Smarca5* cKO mice (Alvarez-Saavedra et al., 2014). In contrast, *Smarca1* showed very weak expression in the VZ and rhombic lip appearing more prevalently after birth in differentiating granule neurons (Alvarez-Saavedra et al., 2014). In the cerebral cortex, mice with a targeted deletion of exon 6 of the *Smarca1* gene (Ex6DEL mice) that encodes the ATP-binding pocket resulted in increased proliferation of intermediate progenitor cells (IPCs) that delayed differentiation and gave rise to animals with a larger brain (Yip et al., 2012). These studies suggest that the ISWI proteins Snf2h and Snf2l play distinct and critical roles in regulating the transition of a proliferating progenitor to a differentiated neuron.

The aim of the current study was to determine the effects of *Smarca1* loss on GNP proliferation and granule neuron differentiation in the developing cerebellum. Moreover, we sought to utilize primary GNP cultures to assess the global changes to the chromatin landscape and transcriptome as a means to define the underlying function of Snf2l during neurogenesis. In this way, we established that GNPs require Snf2l to limit chromatin accessibility at key TSS to promote differentiation. Cultures from the Ex6DEL mice showed an enrichment of chromatin accessibility, particularly at the TSS of genes containing Fos/Jun binding sites that delayed differentiation. Despite the delay in differentiation the Ex6DEL cells utilized a similar genetic program to differentiate that was characterized by increased activation of the ERK pathway.

## Materials and Methods

### Animal Work

The *Smarca1* gene resides on the X chromosome and the generation of the *Smarca1*^Ex6DEL/Y^ male mice have been described previously (Yip et al., 2012). Animals were maintained on an FVB/N background and housed in an animal facility under SPF (specific pathogen-free) conditions on a 12/12 light:dark cycle with water and food *ad libitum.* All animal experiments were approved by the University of Ottawa’s Animal Care ethics committee, with the guidelines set out by the Canadian Council on Animal Care. *Smarca1*^Ex6DEL/+^ female mice were bred with wild-type males and pups were harvested between 4 and 6 days after birth for GNP isolation, or at postnatal day 10 (P10) for RNA isolation.

### GNP Cultures

GNPs were isolated from the cerebella of postnatal day (P)4-P6 pups as previously described by Lee et al. (2009). To increase GNP culture purity, GNPs were passed through a 60–35% Percoll step gradient (Sigma, cat # P4937), wherein GNPs settled at the 35–60% interphase. Cells were plated in serum-free Neurobasal^TM^-A medium with B-27 supplement (Thermo Scientific, cat # 0080085-SA) at a density of 5.5 × 10^5^ cells/well of a 24 well plate coated with 1 mg/ml Poly-D-lysine hydrobromide (Sigma, cat # P6407). A partial media change was performed within a day of isolation and repeated every 48 h. GNP viability was assessed using the Beckman Coulter Vi-CELLTM XR Cell Viability Analyzer default cell counting system (cell size 5–50 μM). Cell viability was assessed by trypan blue dye exclusion method.

Genotyping was performed on genomic DNA from freshly isolated GNP cultures after dissection under the following PCR conditions: a denaturing cycle at 94°C for 2 min, 39 PCR cycles (94°C for 30 s, 60°C for 30 s, 72°C for 45 s) and a final cycle at 72°C for 10 min. A three primer system was used for genotyping with two primers located in the introns flanking exon 6 (Smarca1Intron5For: 5′-CCTGGGCTGGAACCATGATC-3′ and Smarca1Intron6Rev: 5′-GTATGGACAAGTGTGTGAAGCC-3′) and a third primer located within Exon 6 (Smarca1Exon6Rev: 5′-CCATGTGGGGTCCAGGAATG-3′). PCR conditions result in the amplification of only the smaller WT product of 509 bp (Intron5-Exon6; the larger 1108 bp Intron5-Intron6 product is undetectable). The Ex6DEL product is 450 bp and the PCR reactions were electrophoresed on a 1.5–2% agarose gel for genotype analysis.

### BrdU-Pulse Labeling and Immunostaining

Granule neuron precursors cultured on coverslips (*n* = 4) were pulse labeled by adding BrdU (50 μM; Sigma, cat # B-5002) directly to the culture for 2 h. For BrdU immunodetection, cells were fixed (2% PFA, 10 min, RT) and permeabilized (PBS with 0.03% triton-X 100, 10 min at RT) and then were subjected to a DNA hydrolysis incubation (2.5 N HCl, 10 min, RT) prior to immunostaining. For immunostaining, cells were fixed to coverslips and permeabilized as described above. Cells were blocked in 10% horse serum (Life Technologies, cat # 26050-088) in TBST with BSA for 1 hr at RT. Primary antibodies rabbit anti-NF200 (1:500, Sigma, N4142); rabbit anti-Ki67 (1:500, Abcam, ab16667); mouse anti-BrdU (1:500, BD Bioscience, 347580); or rabbit anti-GFAP (1:500, Stem Cell Technologies, 01415) were diluted 1:500 in blocking solution and incubated on the coverslips overnight at 4°C. Secondary Alexa Fluor^®^ (Jackson Immunoresearch) antibody was diluted 1:4000 in PBS and applied for 30 min RT prior to 5 min in a 1:10000 bis-benzimide-Hoescht 33342 (Sigma, United States, cat#B2261) solution in PBS. Coverslips were mounted onto slides with mounting medium (Agilent Technologies, cat # S3023). Coverslips were imaged with an Axio Imager M1 microscope (Zeiss) using either 20X or 40X objectives. Images (6 per time point for each replicate) were prepared with Fiji software^1^ (version 2.0.0). All BrdU-positive cell counts were performed relative to DAPI-labeled nuclei and statistically analyzed by two-way ANOVA from 3-replicate experiments using Excel software. Statistical significance was assumed when the *p*-value was less than 0.05. *p*-values were annotated on the figures as follows: ^∗^*p* < 0.05; ^∗∗^*p* < 0.01; ^∗∗∗^*p* < 0.001.

### Immunoblotting

Cerebellar extracts were quickly dissected from individual pups and then homogenized in ice-cold lysis buffer supplemented with protease inhibitor cocktail (Sigma, United States; P8340) and phosphatase inhibitor cocktail (Fisher, cat # 78441), and then incubated for 10 min at 4°C with gentle mixing. Cultured GNP cell lysates were prepared similarly following resuspension in PBS with a cell scraper. After pre-clearing by centrifugation (5 min at 17,000 × *g*), proteins were quantified by the Bradford method (BioRad, cat # 500-00006). Protein samples were resolved on sodium dodecyl sulfate polyacrylamide gels under denaturing conditions or using Bis-Tris 4–12% gradient gels (NuPage, Invitrogen, United States; cat # NP0007) and blotted onto PVDF membranes (BioRad, cat # 162-0177) by wet transfer for 1 hr at 90V. Membranes were blocked (45 min, room temperature) with 5% skim milk in TBST and incubated (4°C, overnight) in primary antibody [rabbit anti-Snf2l (1:2000, Abcam, ab37003); rabbit anti-Snf2h (1:2000, Abcam, ab72499); rabbit anti-vinculin (1:2000, Abcam, ab129002); rabbit anti-Ki67 (1:2000, Abcam, ab16667); mouse anti-NeuN (1:2000, Millipore, MAB377); mouse anti-Tuj1 (1:2000, Stem Cell Technologies, 01409); rabbit anti-CECR2 (1:1000; gift from Dr. Heather McDermid, uAlberta); rabbit anti-ERK (1:2000, Santa Cruz, sc154); or mouse anti-pERK (1:500, Santa Cruz, sc7383)]. Membranes were incubated (1 h, RT) with ImmunoPure^®^ HRP-conjugated goat anti-rabbit or goat anti-mouse IgG (H + L) secondary antibodies (1:25000; Pierce, Rockford, IL, United States). Membranes were washed 3 × 10 min in TBST after antibody incubations, and the signal was detected using the Pierce Supersignal West Fempto chemiluminescence substrate (ThermoFisher Scientific, cat # 34095). At least 2 separate Western blots were quantified using ImageJ software for quantitation (Schneider et al., 2012).

### Co-immunoprecipitation (co-IP)

Each co-IP was prepared with 500 μg of P21 cerebellar protein lysate (described above), 1 μg antibody, protease inhibitors and lysis buffer to bring the final co-IP volume to 500 μL, which was then rocked overnight at 4°C. Protein A/G magnetic beads (Bioclone Inc, #MA-102) was rinsed twice on a magnetized stand (Thermo Fisher) with two volumes of non-denaturing lysis buffer. Thirty μL bead slurry was then added to each co-IP reaction for capture on a rocking platform at 4°C for 1 h. Beads were washed 5 times in 1 mL 0.3% triton-X in PBS, with each wash being performed on a rocking platform at 4°C for 5 min. Beads were resuspended in 0.1M glycine (pH 2.5) elution buffer and incubated for 10 min RT prior to recovering eluate. Elution was repeated 3 additional times. Samples were prepared in 1:3 in 1X NuPAGE LDS Sample Buffer and 1.5% β-mercaptoethanol (Sigma, #M7522) prior to immunoblotting.

### qRT-PCR

RNA was isolated from P10 cerebella or cultured GNPs using TRIzol Reagent (Life Technologies, #15596018) according to manufacturer’s instructions. RNA was purified using DNA-*free* kit (ThermoFisher Scientific, AM1906) according to the manufacturer’s instructions. Complementary DNA (cDNA) was generated with the RevertAid First Strand cDNA Synthesis Kit (Thermo Fisher, #K1621) and was carried out in a thermocycler for 5 min at 25°C, 1 h at 42°C followed by 5 min at 70°C. qPCR reactions were prepared by mixing 10 μL Lo-ROX 2X SYBR Master Mix (FroggaBio Inc, #BIO-94020) with 0.5 μM of primers and 200 ng of cDNA. Primer used in this study are listed in Table 1. All reactions were performed in technical triplicates. qPCR was carried out on MicroAmp^®^ Fast Optical 96-well Reaction Plate (Life Technologies, #4346906) and run on 7500 Applied Biosystems^®^ Fast Real-Time PCR System [95°C 5 min, 40 cycles (95°C for 5 s, 60°C for 15 s, 72°C for 20 s), and 72°C for 5 min]. Relative fold change was normalized using two controls (18S rRNA and GAPDH) and calculated using ΔΔCt method. Statistical significance was determined using Student’s *t*-test.

**TABLE 1:** List of qPCR primers used for validation of differential gene expression.

BDNF	F: 5′-AGCCTCCTCTTCTCTTTCTGCTGGA-3′ R: 5′-CTTTTGTTGTCTATGCCCCTGCCTT-3′
Sox2	F: 5′-TGCTCAAGATCAAATGGC-3′ R: 5′-GGACTTTTGACCCAGTG-3′
TH	F: 5′-TCCCCAAGGTTCATTGGACG-3′ R: 5′-GGTACCCTATGCATTTAGCT-3′
c-Fos	F: 5′-GGGGACAGCCTTTCCTACTA-3′ R: 5′-CTGTCACCGTGGGGATAAAG-3′
FosB	F: 5′-AAGTGTGCTGTGGAGTTC-3′ R: 5′-ATGTTGGAAGTGGTCGA-3′
Fosl2	F: 5′-CCAGCAGAAGTTCCGGGTAG-3′ R: 5′-GTAGGGATGTGAGCGTGGATA-3′
JunB	F: 5′-TCACGACGACTCTTACGCAG-3′ R: 5′-CCTTGAGACCCCGATAGGGA-3′
Jun	F: 5′-CCTTCTACGACGATGCCCTC-3′ R: 5′-GGTTCAAGGTCATGCTCTGTTT-3′
c-Jun	F: 5′-ACGACCTTCTACGACGATGC-3′ R: 5′-CCAGGTTCAAGGTCATGCTC-3′
JunD	F: 5′-CCAGGTTCAAGGTCATGCTC-3′ R: 5′-AGCCCGTTGGACTGGATGA-3′
Ets-1	F: 5′-AAAGAGTGCTTCCTCGAGCT-3′ R: 5′-AGGCTGTTGAAGGATGACTG-3′
Edn-1	F: 5′-TTTTTCCCCACTCTTCTGACCC-3′ R: 5′-AGTCCATACGGTACGACG-3′
Ednra	F: 5′-CAACTGTGTCTAGGAGGTGGGG-3′ R: 5′-ATGGTCAGCCAAAAGTATGCCG-3′
Ednrb	F: 5′-TTGCTCGCAGAGGACTGGCCA-3′ R: 5′-AAGCATGCAGACCCTTAGGGG-3′
Fgf-3	F: 5′-TCCACAAACTCACACTCTGC-3′ R: 5′-GAACAGCGCCTATAGCATCC-3′
Fgf-5	F: 5′-AACTCCTCGTATTCCTACAATCC-3′ R: 5′-CGGATGGCAAAGTCAATGG-3′
18S	F: 5′-TGTCTCAAAGATTAAGCCATGC-3′ R: 5′-GCGACCAAAGAAACCATAAC-3′
Gapdh	F: 5′-ATCCACGACGGACACATTGG-3′ R: 5′ -CAACGACCCCTTCATTGACCTC-3′

### RNA-seq Analysis

RNA was isolated from GNPs (∼3 × 10^6^ cells) using TRIzol (Life Technologies, #15596018) according to manufacturer’s instructions. RNA cleanup was performed with PureLink RNA Mini Kit (Thermo Fisher, #12183020) with in-column DNaseI digestion (Thermo Fisher, AM1906). RNA samples (*n* = 3) were sequenced at GenomeQuébec (Montréal). RNA integrity was confirmed upon arrival by Bioanalyzer prior to cDNA library generation. Paired RNA-seq was performed on HiSeq4000 PE 100 bp lane and a minimum of 35 million reads was obtained per sample. Quality control (QC) on raw.fastq files was carried out with FastQC (version 0.11.5). Reads were pseudoaligned to GRCm38 (release 88) by kallisto (version 0.44.0; bootstraps = 50) and quantified with Sleuth (version 0.29.0). Differentially expressed genes (DEGs) set a fold-change threshold of ±1.5 with qval ≤0.05. Heatmaps were generated with pheatmap (version 1.0.10) and RColorBrewer (version 1.1-2). Gene Ontology (GO) enrichment analysis^2^ established enriched biological process terms (significant enrichment set at fold enrichment ≥ 1.5 and FDR ≤ 0.05). oPOSSUM was used to identify the transcription factor binding sites within promoter sequences of the differentially expressed genes (Kwon et al., 2012). RNA-seq data was deposited into the GEO database with the accession number GSE122173.

### ATAC-seq Sample Preparation

Samples from WT and Ex6DEL GNP cultures [1 days *in vitro* (DIV) and 3DIV; *n* = 2] were prepared according to Buenrostro et al. (2013, 2015) with modification in transposase reaction conditions. Briefly, 50,000 cells were pelleted, resuspended in 50 μL lysis buffer and centrifuged (500 × *g* for 10 min at 4°C). Transposition mix was prepared by combining 25 μL Nextera TD 2X reaction buffer and 5 μL Nextera TDE1 Tn5 Transposase (Illumina, #FC-121- 1030) to 20 μL nuclease free H2O. Nuclei pellet was resuspended in transposition mix and incubated for 40 min at 37°C. Transposed DNA was purified and eluted using Qiagen MinElute PCR Purification Kit (Qiagen, #28004). The PCR reaction was prepared with 10 μL eluted tagmented DNA, 10 μL nuclease-free H2O, 2.5 μL 25 μM ATAC PCR primers and 2X NEBNext High-Fidelity 2X PCR master mix (New England Biolabs, #M0541S). Amplification and qPCR side reaction was carried out as previously described by Buenrostro et al. (2013, 2015). The amplified library was cleaned with Qiagen MinElute PCR Purification Kit and verified by Bioanalyzer prior to sequencing (Stemcore, Ottawa Hospital Research Institute). Sequencing was performed with Illumina NextSeq500 (PE 75 bp) and an average of 100 million reads were obtained per sample (Stemcore, Ottawa Hospital Research Institute).

### ATAC-seq Analysis

Quality control on raw fastq files was carried out with FastQC (version 0.11.6). Adapters were trimmed using atactk (version 0.1.5) and aligned to mm10 with Bowtie 2 (version 2.3.4). Duplicates were removed with Picard tools (version 2.17.0) and peaks were called using macs2 –shift −100, –ext size 200 in –broad mode (qval < 0.1). BigWig files were prepared for visualization with deepTools2 bamCoverage command and normalized to reads per kilobase per million mapped reads (RPKM) and visualized with IGV (Thorvaldsdottir et al., 2013). DeepTools2 was used to plot read abundance over scaled gene and accessibility heatmaps (Ramirez et al., 2016). Differentially accessible regions (DARs) were established from consensus peaks of merged replicates without summits with DiffBind (version 2.8.0; Ross-Innes et al., 2012). Distribution over genomic features was plotted with ChIPpeakAnno (version 3.14.0; Zhu et al., 2010). ATACseqQC (version 1.2.9; Ou et al., 2018) was used to separate reads based on length. HOMER was used to annotate peaks, as well as to identify and quantify differential motif binding sites on accessible peaks (Heinz et al., 2010). SeqPlots was used to quantify and compare accessible TSS (Stempor and Ahringer, 2016). mESC E14 merged control ATACseq dataset (GEO number GSE98390) was retrieved and analyzed similarly using deepTools2. DAVID was used to identify the biological process, Gene Ontology (GO) functional annotation of ATAC peaks alongside the 0 and 72 h DEGs (Huang da et al., 2009). Dplyr^3^, ggplot2^4^, and Vennerable^5^ were then used to organize and plot results in R (RStudio Team., 2020). Gene Transcription Regulation Database (GRTD) was used to identify the overlap of Fos/Jun/AP-1 binding sites between the DAR and DEG list of the granule neuron progenitor cell population^5^. The ATAC-seq data was deposited into the GEO database with the accession number GSE122172.

## Results

**FIGURE 1:**
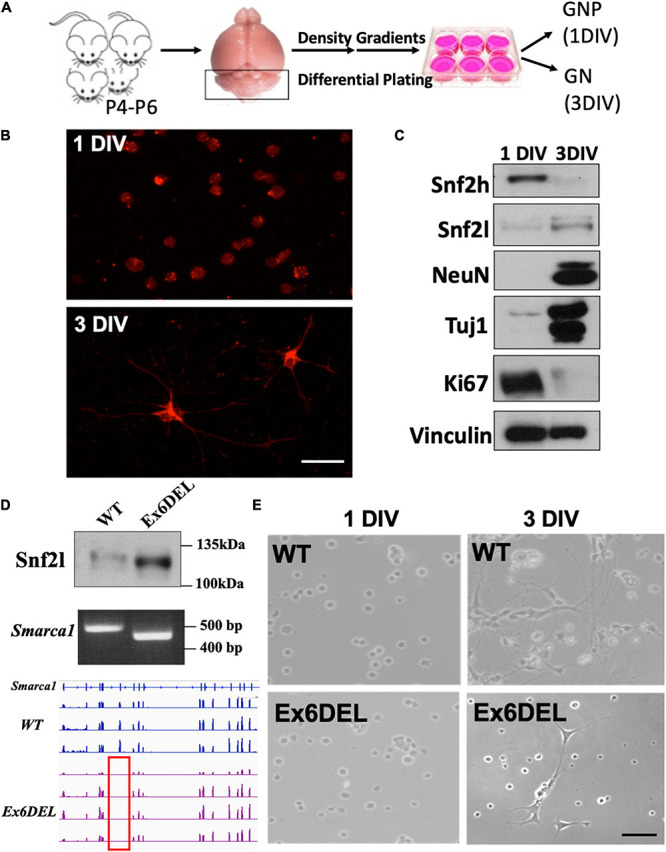
Generation of GNP cultures from the Ex6DEL mice. **(A)** Schematic diagram of the procedure for generating GNP and granule neuron cultures. **(B)** Representative images of WT cultures 1 DIV (top image) and 3 DIV (bottom image) stained with neurofilament-200 (red). Scale bar, 25 μM. **(C)** Protein extracts of primary GNP cultures at I and 3 DIV were immunoblotted for the ISWI proteins (Snf2h, Snf2l), neuronal (Tuj, NeuN) and proliferation (Ki67) markers. Vinculin was used as a loading control. **(D)** Immunoblot (top panel), *Smarca1* genotyping (middle panel), and RNAseq analysis (bottom panel) from WT and Ex6DEL GNP cultures confirmed the loss of exon 6 in the GNP cultures isolated from Ex6DEL mice. The red rectangle outlines the absence of RNAseq reads corresponding to the position of exon 6. **(E)** Phase contrast images of GNP cultures from WT and Ex6DEL mice. Scale bar, 50 μM.

During forebrain development, the *ISWI* homologs *Smarca5* and *Smarca1* (encoding Snf2h and Snf2l proteins, respectively) play essential non-redundant roles. When *Smarca5* is inactivated progenitor expansion is compromised leading to cortical hypoplasia (Alvarez-Saavedra et al., 2019). In contrast, the Ex6DEL mice, containing an internal deletion in the *Smarca1* gene and lacking a functional Snf2l protein present with an enlarged brain due to hyperproliferation of the IPCs (Yip et al., 2012). Taken together, this data suggests that Snf2l and Snf2h have differential effects on progenitor cell homeostasis. *Smarca5* and *Smarca1* are both expressed in the developing postnatal cerebellum, with the early loss of expression of *Smarca5* causing a severe reduction in cerebellar granule neuron progenitor expansion and cerebellar growth (Alvarez-Saavedra et al., 2014). To determine whether the ISWI proteins have antagonistic roles in the cerebellum, we isolated primary cultures of GNPs from WT and Ex6DEL mice to study the role of the Snf2l protein (Figure 1A; Oliver et al., 2005; Bassett et al., 2016). Freshly isolated GNPs from WT spontaneously differentiate over 3 days in culture into granule neurons (GNs) in the absence of Sonic hedgehog protein (Shh) or agonist (Wechsler-Reya and Scott, 1999). The GNP cultures had a high level of purity as we observed < 5% of cells positive for glial acidic fibrillary protein (GFAP; Supplementary Figure 1A). Immunostaining for neurofilament 200 (NF200) of the GNPs from WT suggested a neuronal identity and, by 3 DIV, highlighted the growth of neurite extensions (Figure 1B) that were also readily visible by phase-contrast microscopy (WT, Figure 1E). Immunoblot analysis at 1 DIV and at 3 DIV from WT GNP cultures indicated that Snf2l protein levels increased 3.6-fold with differentiation while Snf2h protein levels decreased by 11-fold (Figure 1C), consistent with our observations in murine cerebellar extracts (Alvarez-Saavedra et al., 2014). The WT cells were also immunoblotted for two pan-neuronal markers (NeuN, Tuj1) and a protein characteristic of cycling cells (Ki67). These blots indicated that GNPs of WT had exited the cell cycle and obtained neuronal identity by 3 DIV (Figure 1C). Next, we isolated GNPs from the Ex6DEL mice to compare their differentiation properties. Immunoblot and PCR genotyping analysis (WT band 508 bp; Ex6DEL band 450 bp) confirmed that the cells were deleted for exon 6 and expressed the internally truncated Snf2l protein (WT protein 122 kDa; Ex6DEL protein 115 kDa; Figure 1D). While the cultures were very similar at 1 DIV, we observed that the majority of the Ex6DEL GNPs had not extended neurites by 3 DIV (Figure 1E) despite insignificant differences in cell viability (Supplementary Figure 1B).

### Ex6DEL GNP Cultures Show Delayed Differentiation Upon Plating

**FIGURE 2:**
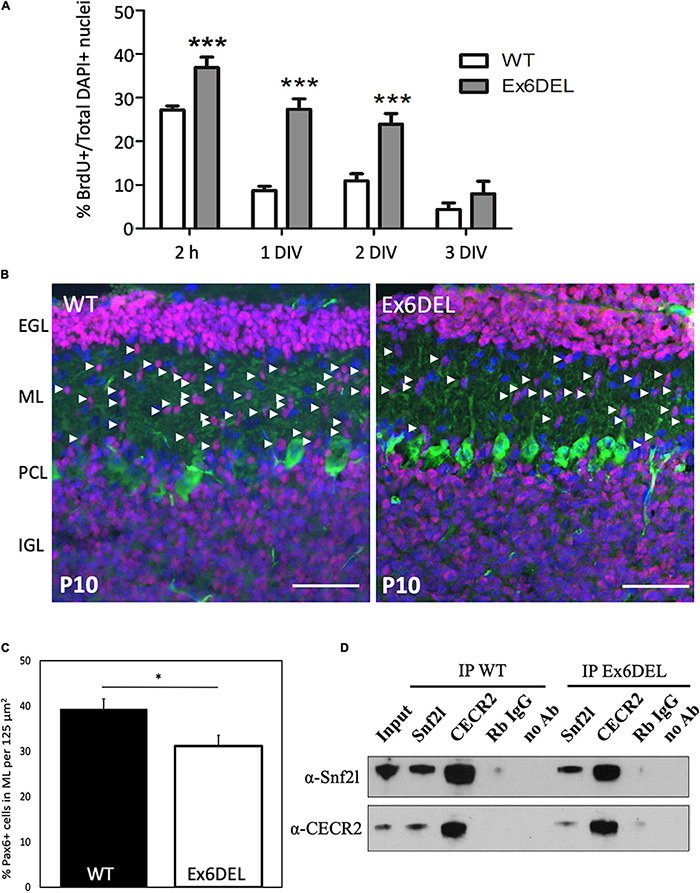
Ex6DEL cultures show delayed differentiation. **(A)** Graph depicting the fraction of BrdU + cells in WT (white bars) and Ex6DEL (gray bars) GNP cultures at the specified times. Cultures were pulsed with BrdU for 2 h prior to harvesting cells for analysis. (*n* = 4 independent experiments; five images per coverslip used for quantification). ***p < 0.001. **(B)** P10 cerebellar sections from WT and Ex6DEL mice stained for committed granule neurons (Pax6, magenta), Purkinje neurons (Calbindin, green) or all cell nuclei (DAPI, blue). Arrowheads indicate the migrating postmitotic granule neurons. EGL, external granule layer; ML, molecular layer; PCL, Purkinje cell layer; IGL, inner granule layer. Scale bar, 50 μM. **(C)** Plot of the percentage of Pax6^+^ cells within the ML. ^∗^*p* < 0.05. **(D)** Immunoblot analysis of cerebellar extracts from WT and Ex6DEL mice co-immunoprecipitated for Snf2l, Cecr2, or a control rabbit antibody (Rb IgG).

Within the developing forebrain of Ex6DEL mice we had previously demonstrated that an increased proportion of IPCs underwent self-renewal and that this in turn delayed cell differentiation (Yip et al., 2012). To assess whether GNPs had a similar fate, we initially immunostained 1 and 3 DIV cultures for Ki67 (Supplementary Figure 2A). GNPs require Shh as a growth mitogen which is released from Purkinje neurons, and since it is not present in the culture media the GNPs typically complete a final mitotic cycle and initiate differentiation upon plating (Wechsler-Reya and Scott, 1999). At 1 DIV, Ki67^+^ cells were present in both WT GNP and Ex6DEL GNP cultures but Ki67^+^ cells were only detected in the Ex6DEL GNP cultures at 3 DIV (Supplementary Figure 2A) suggesting that cell cycle exit was delayed. As such, we incubated GNP cultures with BrdU 2 h before harvesting them at 2 h, 1 DIV, 2 DIV, and 3 DIV after plating to quantify the proportion of cells in S-phase as a second measure of cell proliferation (four independent experiments per timepoint analyzed, five images quantified per coverslip). As expected, the short BrdU pulse labeled 25–30% of cells at 2 h and incorporation of BrdU decreased in the GNPs from WT to less than 10% after 1 DIV (Figure 2A). In contrast, BrdU incorporation in the GNPs from Ex6DEL mice remained at or above 25% at the 1 DIV and 2 DIV timepoints before dropping below 10% at 3 DIV (Figure 2A). This suggests that the Ex6DEL GNPs are delayed in their ability to exit the cell cycle, which is consistent with what we observed *in vivo* in the developing forebrain of Ex6DEL mice (Yip et al., 2012). In the postnatal developing cerebellum, Pax6^+^ GNPs proliferate in the EGL then the postmitotic granule neurons migrate through the molecular layer to complete their maturation in the IGL. We reasoned that the Ex6DEL cerebellum should contain fewer Pax6^+^ cells migrating through the molecular layer at P10 if cell cycle exit was delayed. P10 cerebellar sections from WT and Ex6DEL mice were stained for Pax6 and the number of Pax6^+^ cells within the molecular layer was quantified. This experiment showed a 25% reduction in the number of migrating Pax6^+^ granule neurons in the molecular layer of Ex6DEL mice compared to WT mice (Figures 2B,C). Taken together, these results suggest that Snf2l is required for timely cell cycle exit that delayed but does not impair granule neuron differentiation as indicated by NeuN and Tuj1 staining in both GNP and cortical neuron cultures (Supplementary Figures 2B–D).

The removal of exon 6 maintains the Snf2l open reading frame (ORF) resulting in an internally truncated protein that renders the enzyme unable to bind and hydrolyze ATP, thereby impairing chromatin remodeling activity (Yip et al., 2012). Nonetheless, it remains possible that the Ex6DEL Snf2l protein is incorporated into ISWI complexes such as CERF. These complexes may then maintain their ability to bind chromatin without catalytic remodeling activity, which could contribute to the delayed differentiation of the GNPs. To assess whether the truncated Snf2l protein in Ex6DEL mice can incorporate into an ISWI complex we performed co-immunoprecipitation (co-IP) experiments with CECR2, the partner protein associated with Snf2l in the CERF complex and abundant in the cerebellum (Banting et al., 2005). Cerebellar lysates from WT and Ex6DEL mice isolated at P21 were used for co-IP with anti-Snf2l and anti-Cecr2 antibodies. As indicated in Figure 2D, we detected the intact CERF complex in both WT and Ex6DEL lysates suggesting that the internally truncated Snf2l protein was incorporated into the CERF complex.

### Increased Proliferation Linked to Edn1/Ednra-MAPK-Fos/Jun Pathway

**FIGURE 3:**
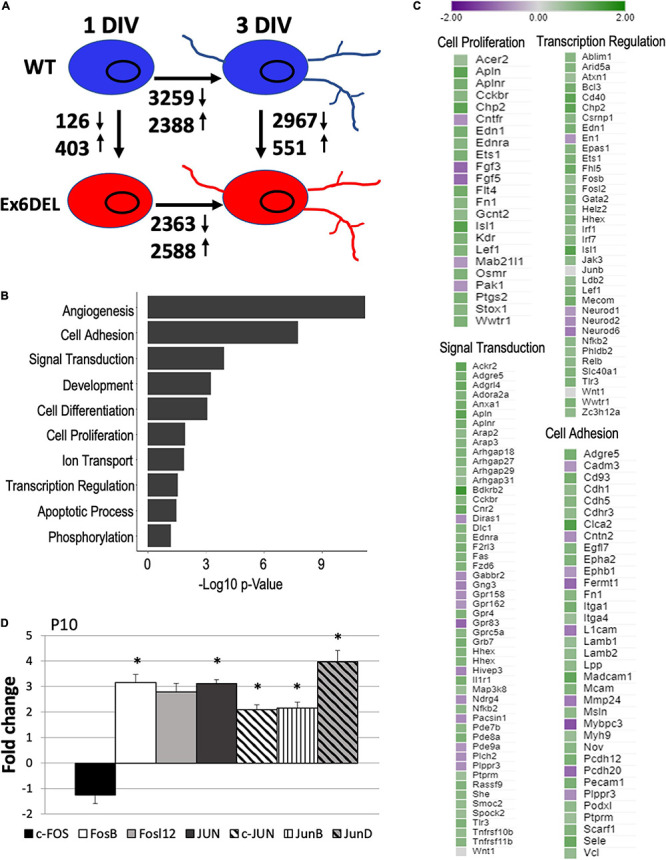
RNAseq analysis of WT and Ex6DEL cultures. **(A)** Schematic diagram showing the numbers of differentially expressed genes (DEGs) between different samples (WT and Ex6DEL) and days in culture. **(B)** GO term analysis of the DEGs observed at 1 DIV. **(C)** DEG lists for some of the top GO terms. **(D)** RT-qPCR validation of Fos/Jun genes from RNA isolated from P10 cerebella. ^∗^*p* < 0.05.

We next performed RNAseq analysis of WT and Ex6DEL cerebellar GNP cultures (∼3 × 10^6^ cells) at 1 and 3 DIV to identify transcriptome differences that might contribute to the delay in cell cycle exit and differentiation. At 1 DIV, we observed 126 downregulated and 403 upregulated genes in Ex6DEL GNPs as compared with WT GNPs (log_2_fold-change threshold ± 0.5 with qval ≤ 0.05; Figure 3A and Supplementary Table 1). GO term analysis of the differentially expressed genes (DEGs) indicated that angiogenesis was the biological process with the most significant change (Figure 3B). It also highlighted a number of biological processes that could mediate GNP homeostasis and contribute to the delayed differentiation including transcriptional regulation, cell signaling, cell adhesion, and cell proliferation (Figure 3B). The specific gene changes we observed that are associated with these four GO terms are highlighted in Figure 3C. It is well known that Shh signaling mediates GNP proliferation (Wechsler-Reya and Scott, 1999), but we did not observe any changes at 1 DIV to the pathway components or downstream effectors (*Gli1*, *N-Myc*, *Math1*, or *Ccnd1*; Supplementary Table 1), except for an increase in *Stox1* (Log_2_FC = 0.80), a gene normally repressed by Shh signaling (Figure 3C). Of note, two members of the mitogenic fibroblast growth factor family (*Fgf3*, Log_2_FC = –1.04; *Fgf5*, Log_2_FC = –1.02) were decreased in expression (Figure 3C). Despite the lack of change in mitogenic pathways affecting GNP proliferation (e.g., Shh and Fgf), we observed altered expression of many genes with known roles in cancer and the epithelial-to-mesenchyme transition (e.g., *Acer2*, Log_2_FC = 0.59; *Apln*, Log_2_FC = 1.20; *Chp2*, Log_2_FC = 1.21; *Cntfr*, Log_2_FC = –0.66; *Pak1*, Log_2_FC = −0.62; and *Wwtr1*, Log_2_FC = 0.89; Figure 3C). In addition, the Wnt receptor *Fzd6* was upregulated (Log_2_FC = 0.84) as were multiple downstream targets of the WNT/β-catenin signaling pathway (*Flt4*, Log_2_FC = 1.00; *Kdr*, Log_2_FC = 0.89; *Lef1*, Log_2_FC = 0.84), including endothelin 1 (*Edn1*, Log_2_FC = 0.93) and its receptor (*Ednra*, Log_2_FC = 0.79) (Figure 3C; Eckey et al., 2012; Katoh, 2018; Adams et al., 2020). The observation suggested that the prolonged proliferative phenotype of the Ex6DEL GNP cultures could arise from activation of the Wnt signaling pathway or dysregulation of genes associated with cancer.

Aside from the genes linked to the GO term Cell Proliferation, we examined our DEG list for changes in *Wnt* and β-catenin (*Ctnnb1*) gene expression but did not observe any changes suggesting that they are not direct transcriptional targets of Snf2l (Figure 3C and Supplementary Table 1). Comparison of our DEG list with known direct and indirect Wnt target genes from two sources (The Wnt Homepage; Boonekamp et al., 2021) revealed increased expression in seven additional Wnt target genes (*Abcb1a*, Log_2_FC = 0.97; *Neurod1*, Log_2_FC = –0.61; *Ptgs2*, Log_2_FC = 0.89; *Fn1*, Log_2_FC = 0.77; and *Plaur*, Log_2_FC = 1.12; *Abcc4*, Log_2_FC = 0.74; *Nes*, Log_2_FC = 0.63) suggesting activation of the Wnt signaling cascade in Ex6DEL cultures. Moreover, several downstream signaling components of the Edn1/Ednra pathway showed increased expression including *Mapk15* (Log_2_FC = 0.87), *Map3k8* (Log_2_FC = 0.61), and *Fos* gene expression (*Fosb*, Log_2_FC = 0.64; *Fosl2*, Log_2_FC = 0.74) suggesting further involvement of this effector pathway (Supplementary Table 1). To assess the Edn1/Ednra-MAPK-Fos/Jun pathway in the Ex6DEL mice we first validated that *Edn1* and both receptors (*Ednra* and *Ednrb*) were upregulated in P10 cerebellum from mutant animals (Supplementary Figure 3A). Similarly, we confirmed a ∼3-fold increase in expression of the *Fosb* (FC = 3.16, *p* = 0.035) and *Fosl2* (FC = 2.79, *p* = 0.39) genes, although Fosl2 did not reach statistical significance (Figure 3D) in the Ex6DEL P10 cerebella, suggesting that the pathway was active both *in vitro* (isolated GNPs) and *in vivo*. However, in the Ex6DEL mice we observed that the expression of *Jun*, *Junb*, and *Jund* were also upregulated in the cerebella, but this was not observed in the Ex6DEL GNP cultures. Taken together, this data suggested that activation of Wnt signaling and dysregulation of the *Edn1*/*Ednra*-MAPK-*Fos*/*Jun* signaling pathway might be a key contributor to the prolonged proliferation of the GNPs isolated from the Ex6DEL mice.

### Increased Promoter Accessibility Associated With Fos/Jun Binding Sites

**FIGURE 4:**
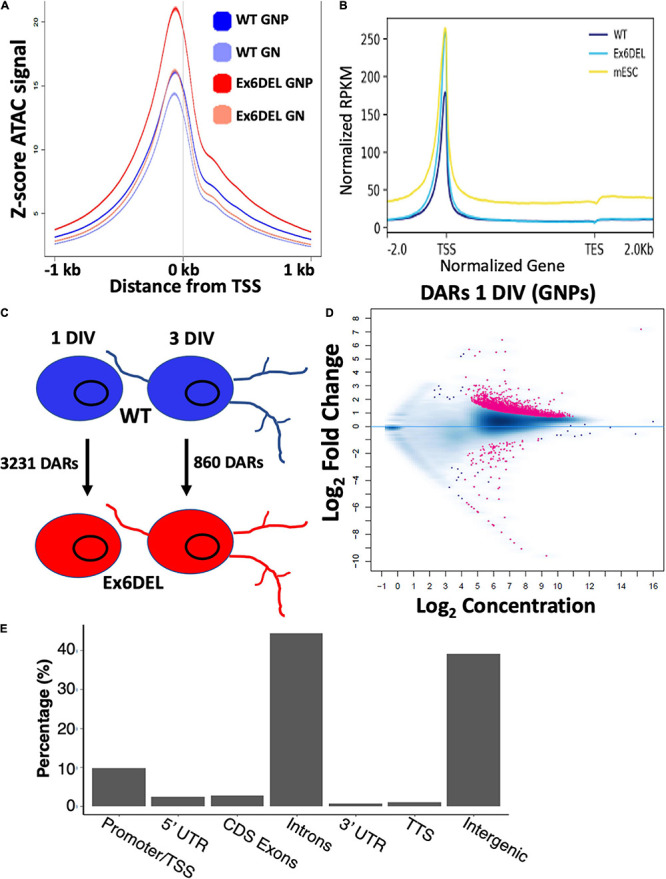
ATACseq analysis of WT and Ex6DEL cultures. **(A)** Compilation of all ATACseq peaks showing enrichment at the TSS under all culture conditions. Mutant cultures at 1 DIV (Ex6DEL GNP) showed the greatest enrichment of peaks at the TSS, followed by Ex6DEL GN (3 DIV), WT GNPs and WT GNs. **(B)** Peaks aligned to a normalized genes showed that Ex6DEL chromatin (light blue) was more accessible than WT chromatin (dark blue) and more similar to the profile of ESCs (yellow). TSS, transcriptional start site; TES, transcriptional end site. **(C)** Schematic diagram showing the numbers of differentially accessible regions (DARs) between WT and Ex6DEL samples at 1 and 3 days in culture. **(D)** MA plot of the 3231 differentially accessible regions. Pink dots represent significant changes in accessibility, with dots above the line representing increased accessibility and below the line decreased accessible regions. **(E)** Plot showing the frequency of DARs at different genomic positions. Promoter/TSS, −1KB to +100 bp; 5′UTR, >100 bp from TSS; TTS, transcriptional termination sites.

The *Drosophila* ISWI protein has been shown to bind near promoters and affect nucleosome positioning adjacent to the transcription start site (TSS) of genes (Sala et al., 2011; Morris et al., 2014). In HeLa cells, both SNF2H and SNF2L were important for organizing nucleosomes adjacent to transcription factor binding sites (Wiechens et al., 2016). As such, we reasoned that loss of Snf2l remodeling activity in GNP cultures would alter the chromatin landscape, particularly at gene regulatory regions. Since we were not able to confirm the validity of the commercial Snf2l antibodies for chromatin immunoprecipitation, it prompted us to employ ATAC-seq to map modifications in chromatin accessibility. ATAC-seq takes advantage of a hyperactive Tn5 transposase which can insert itself and add sequencing primers to sufficiently accessible DNA from nucleosome free regions (NFR) to polynucleosomes (Buenrostro et al., 2013, 2015). We performed ATAC-seq on WT and Ex6DEL GNPs (1 DIV) and GNs (3 DIV) and binned reads according to size, categorizing them as either nucleosome-free (NFR), mono-, di-, or tri-nucleosome reads. Normalized accessibility of WT and Ex6DEL cultures aligned over an averaged gene showed an increased number of reads at the TSS in Ex6DEL samples compared to WT cultures (Figure 4A), most notably corresponding to NFR reads (both time points) and for mono- and di-nucleosome reads at 1 DIV that suggests there is increased accessibility and reduced nucleosome density at the TSS (Supplementary Figure 3B). Progressive chromatin condensation accompanies progenitor cell commitment toward a specialized cell fate. The increased accessibility observed at the TSS in the Ex6DEL progenitors compared to the WT cultures at 1 DIV led us to explore whether the Ex6DEL chromatin accessibility was similar to that of an earlier lineage cell type. To this end, we added a publicly available ATAC-seq E14 mouse embryonic stem cell (mESC) dataset (GEO GSE98390) to our analysis. Interestingly, the mESC ATAC-seq accessibility reads aligned most closely to those of the Ex6DEL GNPs (Figure 4B and Supplementary Figure 4).

**FIGURE 5:**
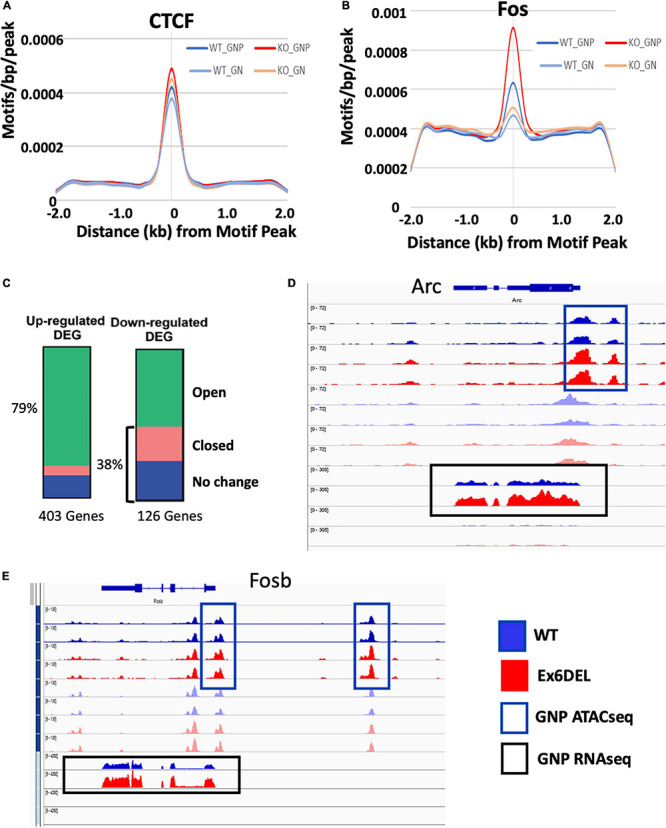
Accessible regions are enriched for Fos/Jun binding sites. ATACseq peak alignment with binding sites for CTCF **(A)** or Fos **(B)** showed enrichment in the Ex6DEL samples for Fos binding sites but not CTCF. **(C)** ATACseq peaks at 1 DIV were assigned to the nearest gene and then cross referenced to the up- and down-regulated genes to generate the bar graphs. Open (green) closed (pink) or chromatin regions showing no change (blue) are shown. **(D,E)** ATACseq and RNAseq reads shown in IGV browser format for the *Arc* **(D)** and *Fosb* **(E)** genes. The GNP ATACseq tracks are shown in the blue box. The GNP RNAseq is shown in the black box. The unboxed tracks correspond to ATACseq and RNAseq from GNs.

We next compared the number of differentially accessible regions (DARs; FDR < 0.05) between WT and Ex6DEL cultures, identifying 3231 DARs at 1 DIV and 860 DARs at 3 DIV (Figure 4C). We focused on the large number of DARs at 1 DIV to determine if these might contribute to the delay in cell differentiation that we observed in the Ex6DEL cultures. Initially, we examined whether the DARs were associated with an increase or a decrease in accessibility. We observed a large increase in the number of accessible chromatin domains (3118), while far fewer DARs (113) showed a reduced level of accessibility within the Ex6DEL cultures compared to the WT cultures (Figure 4D). Overall, the distribution of DARs demonstrated that the majority of accessible regions were located in introns (44%) and intergenic regions (39%) (Figure 4E). The significance of these accessible regions remains to be determined. Nonetheless, an enrichment of 315 DARs was observed at promoter/TSS regions, which were defined as peaks mapped between −1 kb and + 100 bp of a TSS (Figure 4E and Supplementary Table 1); a finding consistent with previous studies (Sala et al., 2011; Morris et al., 2014). Since previous work has indicated that SNF2H and SNF2L organize nucleosomes adjacent to transcription factor binding sites (Wiechens et al., 2016), we examined the 315 promoter/TSS DARs for enrichment of transcription factor binding sites. We first examined CTCF since SNF2H but not SNF2L was required to maintain CTCF occupancy at its binding sites (Wiechens et al., 2016). Similar to that study, we did not observe an enrichment in CTCF motifs in the promoter DARs (Figure 5A). However, we did observe enrichment of 38 transcription factor motifs including Fos-binding motifs (Figure 5B and Supplementary Table 1), which is interesting given the upregulation of *Fosb* and *Fosl2* that we observed at the 1 DIV time point. Indeed, 97 of the 315 promoter DARs contained a Fos/Jun/AP-1 transcription factor binding site within the accessible region (Supplementary Table 1).

Next, we examined the frequency of DARs associated with the DEGs at 1 DIV. All DARs were linked to a gene based on map position to the nearest gene. Overall, 79% of the upregulated genes contained an open DAR associated with them, although only 25 DEGs had a DAR located within the promoter/TSS region (Figure 5C and Supplementary Table 1). Of the 25 genes with a promoter/TSS DAR, thirteen genes (*Akap2*, *Arap2*, *Arc*, *Bhlhe40*, *Col4a2*, *Csrnp1*, *Emp1*, *Ets1*, *Gcnt2*, *P2rx6*, *Wnt9a*, *Wwtr1*, and *Zc3h12a*) also contained a Fos/Jun/AP-1 binding site and were increased in expression (Supplementary Table 1). These genes also contained binding motifs for *Mzf1*, *Klf4*, and *Sp1* transcription factors within the promoter DAR (Supplementary Figure 5).

Analysis of mapped IGV (integrated genome viewer) tracks showed a clear increase in ATAC peaks and RNAseq reads for the 13 genes with a Fos/Jun binding site in the promoter/TSS DAR, as shown for the *Arc* gene (Figure 5D; additional genes shown in Supplementary Figures 6A,B). However, *Fosb* and *Fosl2* upregulation was not associated with a promoter/TSS DAR despite an apparent increase in chromatin accessibility (Figure 5E and Supplementary Figure 6C). Similarly, the IGV tracks for *Mapk15*, *Edn1* and *Ednra* showed increased expression in the Ex6DEL cultures but the increased chromatin accessibility at the promoter/TSS region did not reach the statistical threshold cutoff for a DAR (Supplementary Figures 6D–F). Taken together, we observed a good correlation between increased chromatin accessibility and upregulated gene expression with a subset of genes displaying *Fos*/*Jun* dysregulation.

In contrast to the upregulated genes, 38% of the downregulated DEGs were associated with a DAR that had reduced accessibility or showed no change in chromatin accessibility (Figure 5C). In addition, for genes that showed reduced accessibility we did not observe any overlap with the promoter/TSS DAR list suggesting that downregulation was not associated with altered chromatin structure at the promoter. Surprisingly, 61% of DARs linked to downregulated genes showed enhanced accessibility, which reflects the large number of accessible DARs in introns although the significance of these chromatin changes remains to be determined.

### Ex6DEL Cultures Show Increased Activation of Erk Signaling

**FIGURE 6:**
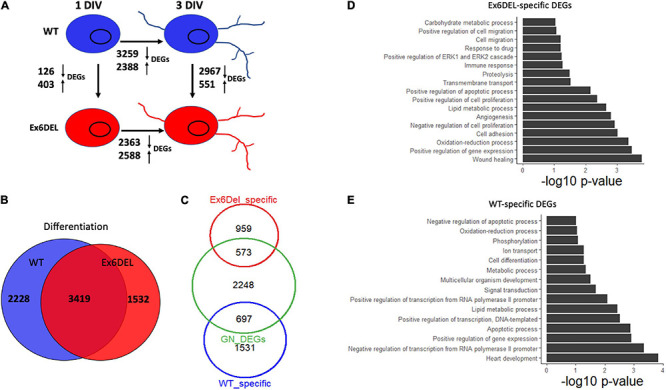
Ex6DEL cultures are delayed in differentiation. **(A)** Schematic diagram showing the numbers of differentially expressed genes (DEGs) between different samples (WT and Ex6DEL) and days in culture (same as Figure 3A). **(B)** Venn diagram showing overlapping differentially expressed genes in WT or Ex6DEL cultures during differentiation (gene expression comparison between I and 3 DIV). **(C)** Venn diagram highlighting that only 1/3 of the uniquely expressed genes from **(B)** (Ex6DEL specific: 1532; WT-specific: 2228) remain differentially expressed at 3 DIV. Green circle comprises the 2967 down- and 551 up-regulated DEGs between WT and Ex6DEL cultures. **(D,E)** GO term analysis of the DEGs at # DIV that were specific to the Ex6DEL **(D)** or WT **(E)** cultures.

Despite the delay exiting the cell cycle, GNPs from the Ex6DEL cultures initiate the differentiation program. We compared the differential gene expression between 1 DIV and 3 DIV timepoints for both WT and Ex6DEL cultures to determine whether the process of differentiation results in similar gene set changes. In this regard, we observed a similar number of genes differentially expressed from 1 DIV to 3 DIV, with 5647 DEGs changing in WT cultures and 4951 DEGs altered in the Ex6DEL cultures (Figure 6A). Moreover, 3419 genes were common to both cultures (WT: 60.5% of DEGs; Ex6DEL: 69% of DEGs) suggesting that the differentiation program proceeds along a similar path regardless of Snf2l status (Figure 6B). There were 2228 and 1532 genes in WT and Ex6DEL cultures, respectively, that were specific to the differentiation of the individual cultures. We reasoned that many of these gene expression differences could reflect the overall lag in differentiation occurring in the Ex6DEL cultures while others could represent Snf2l-specific changes.

Next, we examined the differences in gene expression at 3 DIV to determine how similar or different the GNP cultures were 3 days after plating. This analysis demonstrated 3518 DEGs between the WT GNs and Ex6DEL GNs at 3 DIV (Figure 6A). Given the lag in differentiation of the Ex6DEL cultures, we compared how many of the DEGs between 1 DIV and 3 DIV were resolved by the third day in culture. In both sets of cultures approximately 2/3 of DEGs were resolved (60%, 1531/2228 WT DEGs; 63%, 959/1532 Ex6DEL DEGs; Figure 6C). Alternatively, one-third of the Ex6DEL (573 DEGs) and WT (697 DEGs) remain altered in the 3 DIV cultures, while 2248 “new” genes (GN DEGs) become differentially expressed (Figure 6C). Finally, we performed GO analysis on these clusters of DEGs identified at 3 DIV (i.e., Ex6DEL-specific, WT-specific, GN DEGs). Of the 573 DEGs that were considered to be specific for Ex6DEL 3 DIV cultures, notable GO terms showed changes in cell proliferation, cell migration, cell adhesion, and regulation of the ERK1/ERK2 pathway (Figure 6D). Notable GO terms for the 697 DEGs specific for WT 3 DIV cultures included positive regulation of transcription & gene expression, cell differentiation, multicellular organism development (Figure 6E). Very similar GO terms were also identified for the novel 2248 DEGs at the 3 DIV timepoint suggesting that these latter gene sets are representative of a more mature differentiated state (Supplementary Figure 7).

**FIGURE 7:**
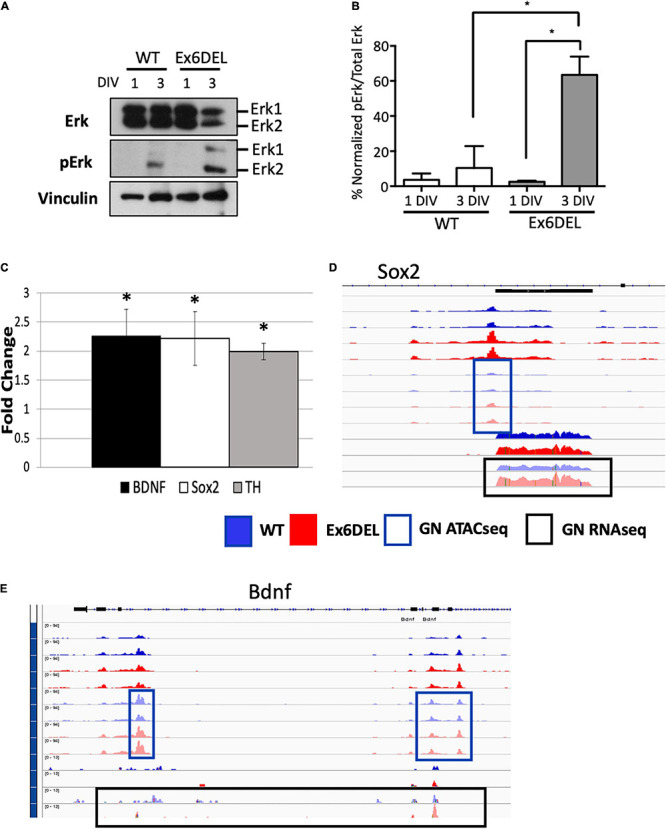
Granule neuron precursor differentiation is associated with ERK signaling activation. **(A)** Immunoblots from WT and Ex6DEL GNP cultures (1 and 3 DIV) for Erk and phospho-Erk (pErk). Vinculin serves as a protein loading control. **(B)** Quantification of the immunoblots in A showing the normalized change in Erk activation (pErk1 + pErk2 level) to total Erk protein level. ^∗^*p* < 0.05. **(C)** RT-qPCR validation of the expression of downstream target genes of ERK/Fos/Jun signaling. ^∗^*p* < 0.05. **(D)** Genome browser views of the *Sox2* **(D)** and *Bdnf* **(E)** genes. The blue boxes highlight the ATACseq peaks and the black boxes mark the RNAseq reads at 3 DIV. All unboxed tracks correspond to the I DIV time.

As such, we focused on the Ex6DEL-specific dataset and, more specifically, the DEGs within the GO term “regulation of ERK1/ERK2 pathway” to identify a potential pathway critical for GN differentiation of the Ex6DEL cells. In this regard, we performed immunoblots for the Erk1/2 proteins and their phosphorylated isoforms. GNPs were cultured from WT and Ex6DEL mice and protein extracts isolated at 1 DIV and 3 DIV for analysis. Since Erk1 and Erk2 exhibit functional redundancy we quantified global Erk phosphorylation (normalized pErk1 and pErk2/Erk1 and Erk2 levels) and not isoform specificity as a measure of pathway activation (Busca et al., 2016). In the WT samples the total Erk protein level dropped by 40% in the 3 DIV sample compared to the 1 DIV sample, but the activation increased ∼3-fold (3.6% at 1 DIV; 10.4% at 3 DIV). It should be noted that the activation was primarily through phosphorylation of Erk2 (Figures 7A,B). In the Ex6DEL cultures we observed a similar drop in total Erk protein levels (∼50%) at 3 DIV but pathway activation was significantly increased (24-fold; 2.6% 1 DIV; 63.4% 3 DIV; Figures 7A,B).

To determine if the Erk pathway is altered in the Ex6DEL mice, we performed RT-qPCR for three downstream Erk target genes, using RNA isolated from cerebellar tissue at P10 from Ex6DEL mice and control littermates. Consistent with increased activation of the Erk1/2 pathway in Ex6DEL 3 DIV cultures we observed increased expression of all three downstream target genes, namely *Sox2*, *Bdnf*, and *Th* in the Ex6DEL cerebellum (Figure 7C). We also examined whether the transcriptional changes in the mice were duplicated in the cultures by assessing the chromatin accessibility and transcript reads of these target genes. For both *Sox2* and *Bdnf* we observed a slight increase in accessibility although none of the accessible regions reached the significant threshold to be considered a DAR (Figures 7D,E). Moreover, increased transcript reads were only observed for *Sox2* (Figure 7D) but not for *Bdnf* (Figure 7E) in the culture experiments.

## Discussion

The Ex6DEL mice contain an internally truncated Snf2l protein that lacks the ability to bind and hydrolyze ATP thereby rendering it unable to remodel nucleosomes. We have shown that the truncated Snf2l protein can assemble into ISWI complexes (e.g., CERF) and thus, it likely retains some ability to be recruited to its genomic targets (e.g., promoter/TSS sites). GNPs isolated from the Ex6DEL mice were mildly impaired in their ability to exit the cell cycle, thus delaying differentiation when plated in culture. The chromatin landscape of the Ex6DEL GNP cultures showed a general increase in accessibility at TSS that was more similar to that of mESCs than to control GNP cultures. Over 96% of the DARs between WT and Ex6DEL GNP cultures showed increased accessibility. Of those located in promoter/TSS regions we identified an enrichment for Fos/Jun binding sites suggesting that Snf2l remodeling normally decreases accessibility at these sites to facilitate differentiation. The increased accessibility was correlated with increased gene expression with ∼80% of the 529 DEGs, consistent with a role for Snf2l in repressing gene expression during cerebellar development. Collectively, the Ex6DEL cultures either represent a “less differentiated” or committed GNP that does not immediately exit the cell cycle upon plating, or alternatively, the culture contains a greater proportion of a distinct progenitor cell type that alters the differentiation kinetics of the culture. Regardless, the enhanced proliferation index was transient and the Ex6DEL cultures underwent differentiation showing a 69% concordance with WT cultures in the DEGs associated with this process (i.e., 1 DIV vs. 3 DIV expression changes). Expression differences at 3 DIV highlighted the importance of the ERK signaling cascade for GNP differentiation.

Granule neuron precursors migrate to the EGL from the upper rhombic lip, one of two germinal zones in the developing cerebellum. Within the EGL they respond to Shh released from Purkinje cells to proliferate during the early postnatal period which is critical for cerebellar folia growth. Dysregulation of GNP expansion can result in a hypoplastic cerebellum, as was observed for *Smarca5* cKO mice (Alvarez-Saavedra et al., 2014), or conversely, cause some subtypes of medulloblastoma as shown for activating hedgehog (HH) mutations. We observed a transient maintenance of proliferation in the Ex6DEL cultures that was not associated with alterations in HH signaling suggesting that other mitogenic pathways were active. A previous study demonstrated increased proliferation of HeLa cells after siRNA knockdown of *Smarca1* through the activation of the Wnt signaling cascade (Eckey et al., 2012). The study demonstrated activation of β-catenin using the TOP/FOP flash assay but determined that this was a post-transcriptional effect ruling out SNF2L remodeling of the *CTNNB1* gene. Three of the Wnt target genes upregulated in that study (*EDN1*, *FN1*, *PLAUR*) were similarly altered in our study as were an additional 9 Wnt responsive genes (*Fzd6*, *Flt4*, *Kdr*, *Lef1*, *Abcc4*, *Abcb1a*, *Neurod1*, *Ptgs2*, *Nes*) and the receptor for *Edn1* (*Ednra*). The significance of these changes to proliferation, however, remain to be fully determined. Another similarity between our study and the one by Eckey et al. (2012) was that the GO term analysis indicated that angiogenesis was the biological process with the most significant change. While further work is required to delineate the significance of the specific gene changes, one possibility is that Snf2l is required to maintain repression of genes critical for blood vessel formation.

One of the dysregulated Wnt target genes of interest was the endothelin-1 gene, *Edn1* because it has been shown to be critical for autocrine-mediated neuroprogenitor proliferation within the postnatal subventricular zone of mice (Adams et al., 2020). A similar decrease in proliferation was obtained when one of the endothelin receptors, endothelin b receptor (*Ednrb*) was deleted in mice. Further studies revealed that endothelin signaling activated the Notch pathway to maintain proliferation of the radial glia progenitors (Adams et al., 2020). While a similar Edn1-Ednra autocrine effect could be occurring in the Ex6DEL GNP cultures, we did not observe transcriptional activation of Notch signaling components (e.g., *Jag1*, *Hey1*) as shown in the Adams et al. (2020) study. Despite this difference, endothelin-1 signaling can be transduced through phosphatidylinositol 3-kinase (PI3-K), Wnt, and mitogen-activated protein kinase (MAPK) signaling pathways (Bouallegue et al., 2007; Kristianto et al., 2017), the latter two for which we observed several gene expression differences in the Ex6DEL cultures.

Given the incidence of Wnt activation in medulloblastoma, several studies have examined the requirement for Wnt in GNP proliferation (Lorenz et al., 2011; Pei et al., 2012; Yang et al., 2019). Using different methods to activate Wnt signaling, each study came to the same conclusion, namely that Wnt activation was not mitogenic for GNPs but actually promoted their differentiation. This is opposite to the effect of Wnt signaling in the developing forebrain where b-catenin stabilization resulted in a dramatic increase in progenitor proliferation (Chenn and Walsh, 2002). As such, it seems difficult to reconcile that Wnt activation in the Ex6DEL GNPs is driving their proliferation unless perhaps, we are observing an increased level of a second progenitor subtype in the Ex6DEL cultures. In this regard, a rare population of Nestin-expressing progenitor cells (NEPs) that migrate from the second germinal zone (VZ lining the fourth ventricle) and reside in the deep part of the EGL could be affected by loss of Snf2l function. These NEPs are typically quiescent but can be activated to replenish granule neurons following injury in the postnatal period (Li et al., 2013; Wojcinski et al., 2017, 2019). In such a scenario, the lack of Snf2l in this cell population would alleviate their quiescence and activate their proliferation. As we observed increased *Nes* gene expression, it remains possible that both the activated Wnt signaling signature and the enhanced BrdU incorporation observed in the Ex6DEL cultures result from inappropriate NEP proliferation. Future analyses using a scRNAseq approach should help define progenitor cell type differences within the GNP cultures.

An essential marker of GNPs is the bHLH transcription factor *Atoh1/Math1* which is required for their generation in the rhombic lip, and later for the regulation of primary cilia formation that facilitates proliferation in response to Shh (Ben-Arie et al., 1997; Dahmane and Ruiz i Altaba, 1999; Wallace, 1999; Chang et al., 2019). *Atoh1* also induces the expression of *Zic1*, *En1*, and *Neurod1* to facilitate differentiation, the latter of which has been shown in other experiments to be required for granule neuron differentiation (Miyata et al., 1999; Pan et al., 2009; Iulianella et al., 2019). Examination of the 1 DIV transcriptome indicated that *Neurod1* expression is reduced (Log_2_FC = –0.61) compared to control cultures but at 3 DIV it is differentially increased (Log_2_FC = 0.725) supporting the argument that differentiation is transiently delayed in the Ex6DEL cultures. *Zic1* was increased at the 3 DIV timepoint (Log_2_FC = 0.625) only, while *En1* and *En2* were not altered compared to control cultures.

We also analyzed the gene expression profiles during (1 DIV vs. 3 DIV) and after differentiation (WT vs. Ex6DEL 3 DIV). In this way, we observed that differentiation was associated with similar expression profiles regardless of Snf2l status. This data suggests that Snf2l is not required to initiate GNP differentiation. When we compared expression profiles at 3 DIV we observed activation of the ERK1/2 pathway which we confirmed by immunoblots of the protein extracts from the cultures. Multiple studies have previously shown that a wide range of soluble factors (Glucose, IGF-1, Wnt3) can promote cerebellar granule differentiation through MAPK-ERK1/2 activation (Torres-Aleman et al., 1998; Petersen et al., 2002; Anne et al., 2013; Kronenberg et al., 2020). Our finding that this was specific to the Ex6DEL cultures may simply reflect the delayed differentiation of these cultures. Several of these studies have also linked MAPK-ERK1/2 activation directly to *Neurod1* activity which we mentioned above was upregulated (Petersen et al., 2002; Kronenberg et al., 2020). One study of particular interest demonstrated that Wnt3 promoted GNP differentiation through a non-canonical Wnt signaling pathway that activated the MAPK-ERK1/2 pathway instead of the β-catenin canonical pathway (Anne et al., 2013). Wnt3 (Log_2_FC = 0.690) was also upregulated in the DEGs linked to Ex6DEL differentiation. Regardless, it is clear that GNP cultures isolated from Ex6DEL mice undergo differentiation utilizing a common MAPK-ERK1/2-*Neurod1* pathway previously shown to be important for granule neuron differentiation.

Analysis of *Drosophila* ISWI protein binding sites by ChIPseq showed that the majority of sites mapped to ∼300 bp after the TSS with fewer binding sites located at exons, introns and the 3′-end of several genes (Sala et al., 2011). The role of ISWI binding near the TSS is important for positioning the downstream nucleosomes, particularly the +1 and +2 nucleosome positions (Sala et al., 2011; Wiechens et al., 2016). In mammals, the nucleosome positioning by SNF2H facilitated binding at CTCF binding sites while depletion of SNF2L had minor effects on nucleosome organization at CTCF sites (Wiechens et al., 2016). In addition to CTCF, the authors observed that SNF2H and SNF2L depletion altered the +1 and –1 nucleosome spacing adjacent to 49 different TF binding sites often with contrasting effects on nucleosome spacing, suggesting that they likely have antagonistic effects on TF occupancy (Wiechens et al., 2016). Using an ATACseq approach to define accessible chromatin in GNPs we observed that depletion of Snf2l resulted in increased accessibility at 3118 sites suggesting that Snf2l normally represses access to chromatin. However, the large majority of differentially accessible sites were located in introns and intergenic regions with a smaller fraction (10%) at promoter/TSS regions, which is different than the ChIPseq experiment in *Drosophila* (Sala et al., 2011) and may reflect additional indirect changes in chromatin accessibility. While the nature of the non-coding sites was not examined, a high proportion (30%) of the promoter/TSS DARs contained binding sites for the Fos/Jun transcription factor. This data suggests that Snf2l may have an important role in regulating TF occupancy at Fos/Jun target genes during GNP differentiation. Consistent with this, we found that 25 of the DEGs contained a promoter/TSS DAR and that half of these had Fos/Jun binding sites. Many of the upregulated DEGs showed minor increases in promoter accessibility that did not reach the significance threshold for a DAR (compare *Arc* vs. *Fosb*). It suggests that these genes may contain additional inter- or intra-genic accessible regions that alter access at distal regulatory elements or, change topological chromatin domains to affect expression. Certainly, such changes were underappreciated in the type of analysis performed here but would also represent a unique ISWI function.

In summary, we have shown that the Ex6DEL GNP cultures have a transient delay in cell cycle withdrawal that was also found in the intermediate progenitors of the developing forebrain. GNPs undergo an average of eight divisions during postnatal cerebellar growth producing an average of 250 granule neurons (Iulianella et al., 2019). An extra cycle of proliferation within the GNPs (or IPCs) are more than sufficient to account for the increased brain size observed in the Ex6DEL mice. We suggest that the delay results from a less-restricted chromatin configuration in the Ex6DEL progenitors, allowing continued access to transcription factors such as Fos/Jun. Despite the alterations to the chromatin landscape the Ex6DEL GNPs maintain the ability to differentiate using previously defined pathways.

## Data Availability Statement

The datasets presented in this study can be found in online repositories. The names of the repository/repositories and accession number(s) can be found below: https://www.ncbi.nlm.nih.gov/geo/query/acc.cgi?acc=GSE122172, https://www.ncbi.nlm.nih.gov/geo/query/acc.cgi?acc=GSE122173.

## Ethics Statement

The animal study was reviewed and approved by the University of Ottawa’s Animal Care ethics committee.

## Author Contributions

LG executed all experiments with the technical support of JM, unless stated otherwise. GZ and LG performed the bioinformatic analysis. ST performed the RT-qPCR validation experiments and cerebellar IF staining. DP designed, supervised, and provided funding for the project. LG, GZ, ST, and DJP wrote the manuscript. All the authors contributed to the article and approved the submitted version.

## Conflict of Interest

The authors declare that the research was conducted in the absence of any commercial or financial relationships that could be construed as a potential conflict of interest.

## Footnotes

1 http://gtrd.biouml.org/#!

2 http://www.geneontology.org/

3 https://dplyr.tidyverse.org//

4 https://ggplot2.tidyverse.org

5 https://github.com/js229/Vennerable
