# Supplementary material for: Impaired SNF2L Chromatin Remodeling Prolongs Accessibility at Promoters Enriched for Fos/Jun Binding Sites and Delays Granule Neuron Differentiation
Source: Front Mol Neurosci. 2021 Jul 6;14:680280. doi: 10.3389/fnmol.2021.680280 (PMC8290069; doi:10.3389/fnmol.2021.680280)
Supplement: Supplementary file 2 [file Data_Sheet_1.docx]

**SUPPLEMENTAL INFORMATION**

**Impaired SNF2L chromatin remodeling prolongs accessibility at promoters enriched for Fos/Jun binding sites and delays granule neuron differentiation**

*Laura R. Goodwin, Gerardo Zapata, Sara Timpano, Jacob Marenger, and David J. Picketts*


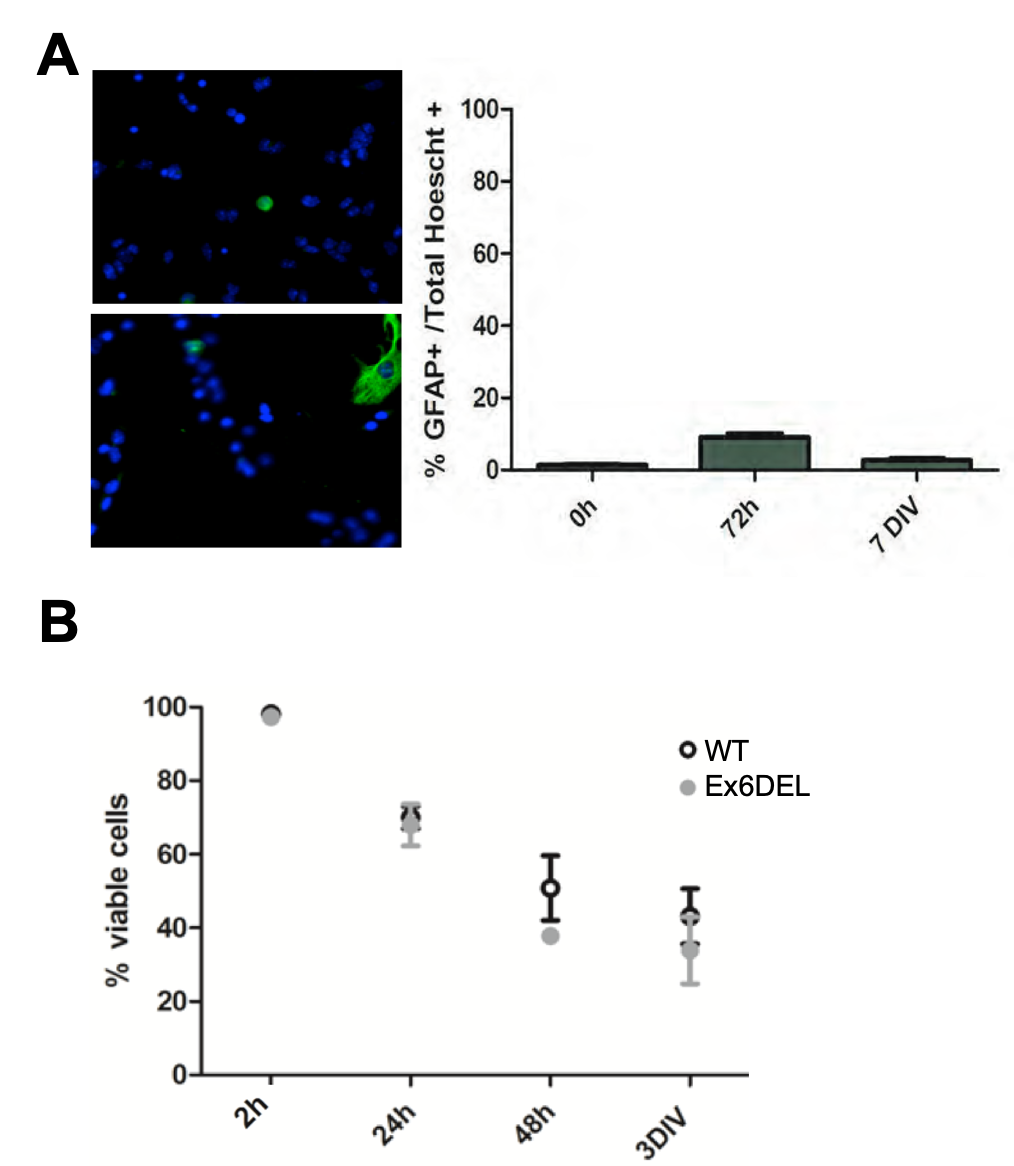


**Supplemental Figure 1. The GNP cultures show no differences in viability and have a low level of astrocyte contamination.** A) Two representative images of WT GNP cultures stained with GFAP (green) to detect contaminating astrocytes. Quantification of the astrocyte+ cells is shown on the right at different times after plating. B) The GNP cultures derived from WT and Ex6DEL mice show no differences in cell viability.


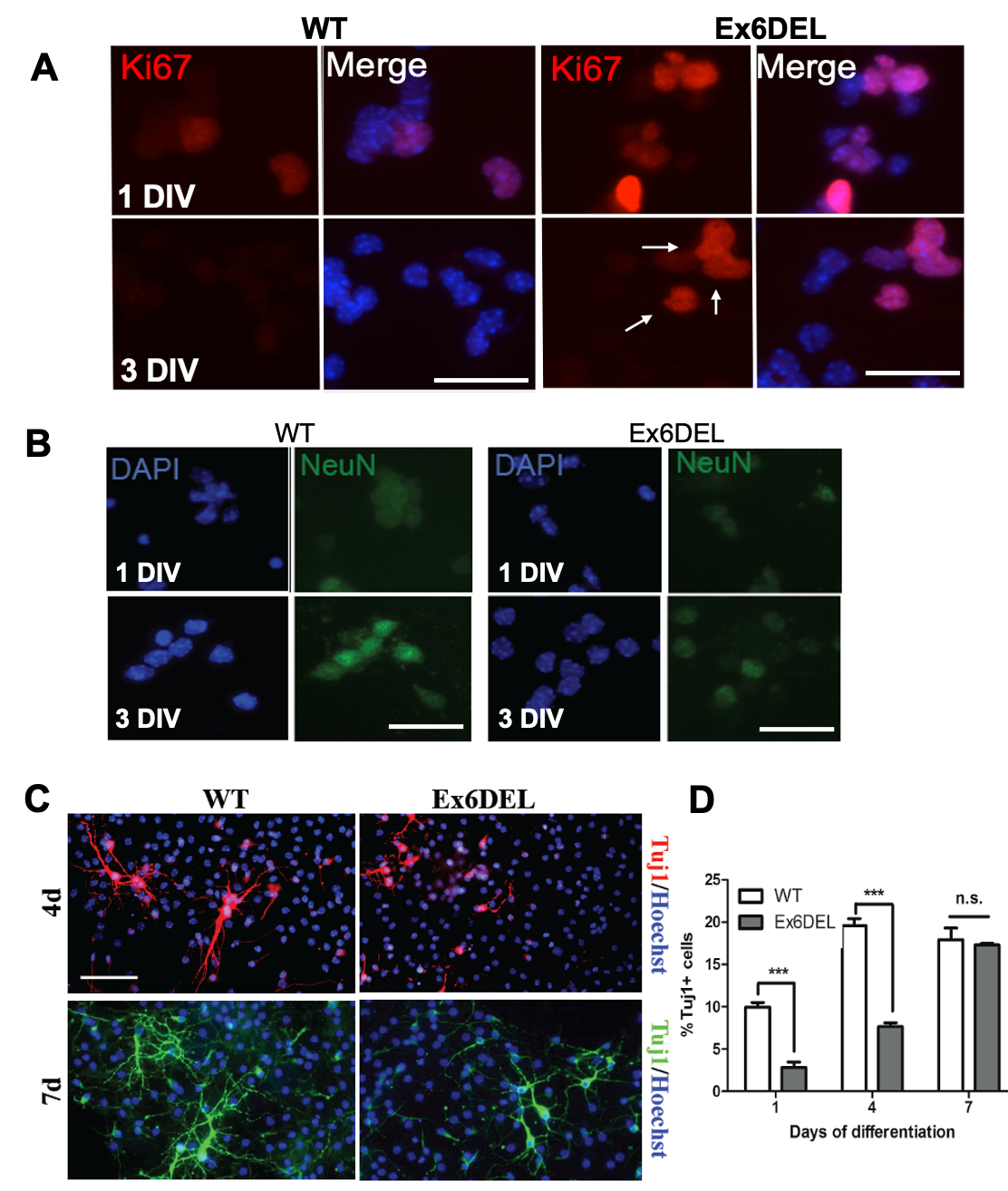


**Supplemental Figure 2. Differentiation of Ex6DEL GNPs**.

Representative images of WT and Ex6DEL GNP cultures at 1 and 3 DIV stained for **A)** the cycling cell marker, Ki67 (red); or for **B)** the differentiation marker NeuN (green) and counterstained with DAPI (blue). Arrows in panel A indicate the persistence of Ki67+ cells at 3 DIV in the Ex6DEL cultures. Scale bar, 25 μM. **C)** Differentiating cortical neurons from WT and Ex6DEL mice were identified by staining with Tuj1+ at 4 days in vitro (red) or 7 days in vitro (green) after inducing differentiation. Note the increased dendritic arborization at 7 days compared to 4 days, and the prominent delay in dendrite formation between Ex6DEL samples and control samples. **D)** Quantification of the percentage of Tuj1+ cortical neurons at 1, 4, and 7 days in culture.


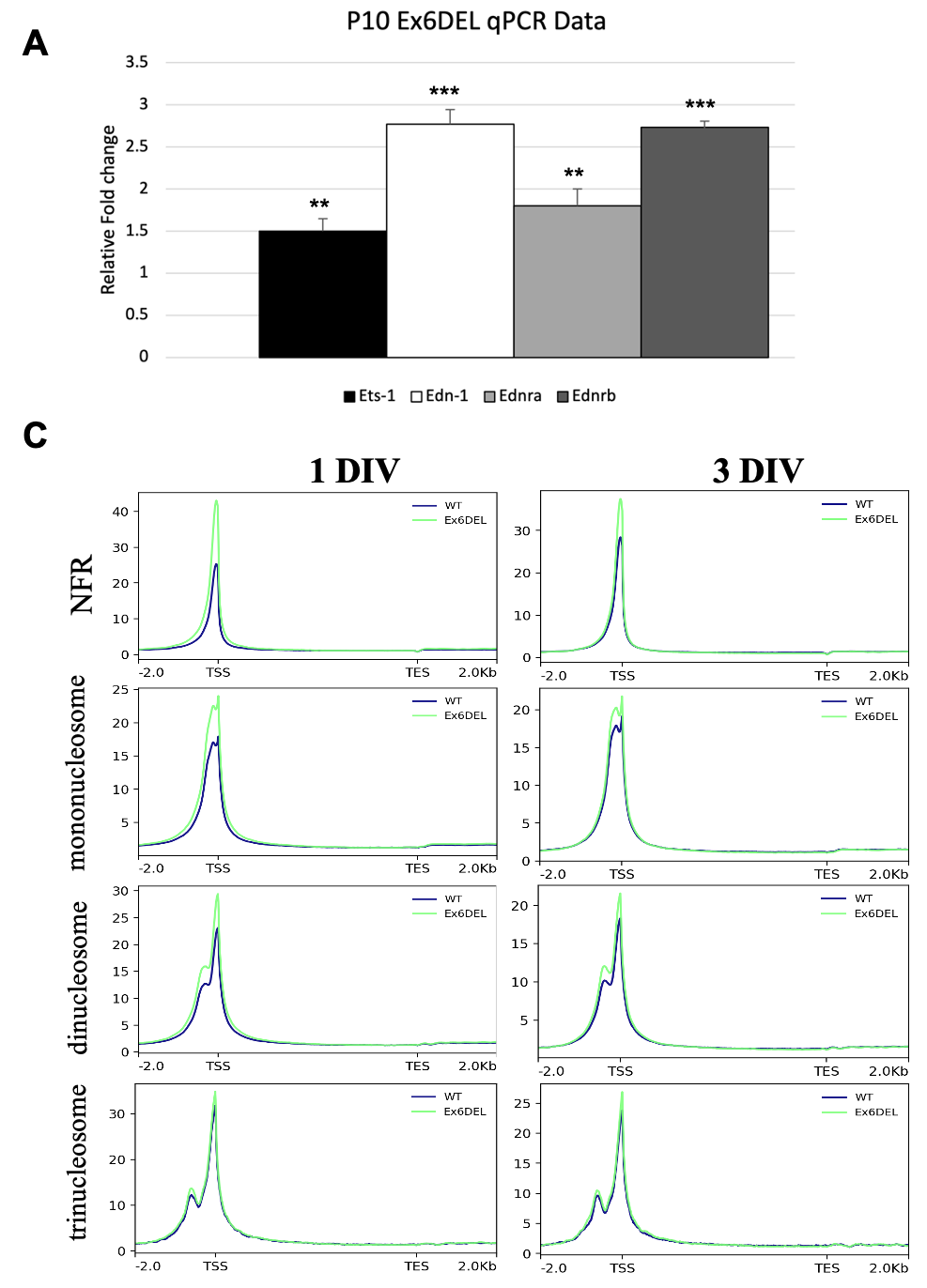


**Supplemental Figure 3. RT-qPCR validation of DEGs and mapped ATACseq reads. A)** RNA isolated from cerebella of P10 WT and Ex6DEL mice was used for RT-qPCR to validate dysregulated genes identified by RNAseq. B) ATACseq reads from 1 and 3 DIV were mapped by size as either nucleosome-free (NFR) or representing mononucleosome, dinucleosome or trinucleosome fragments. Reads were then mapped to a normalized gene for comparison of WT (blue) or Ex6DEL (green) cultures.


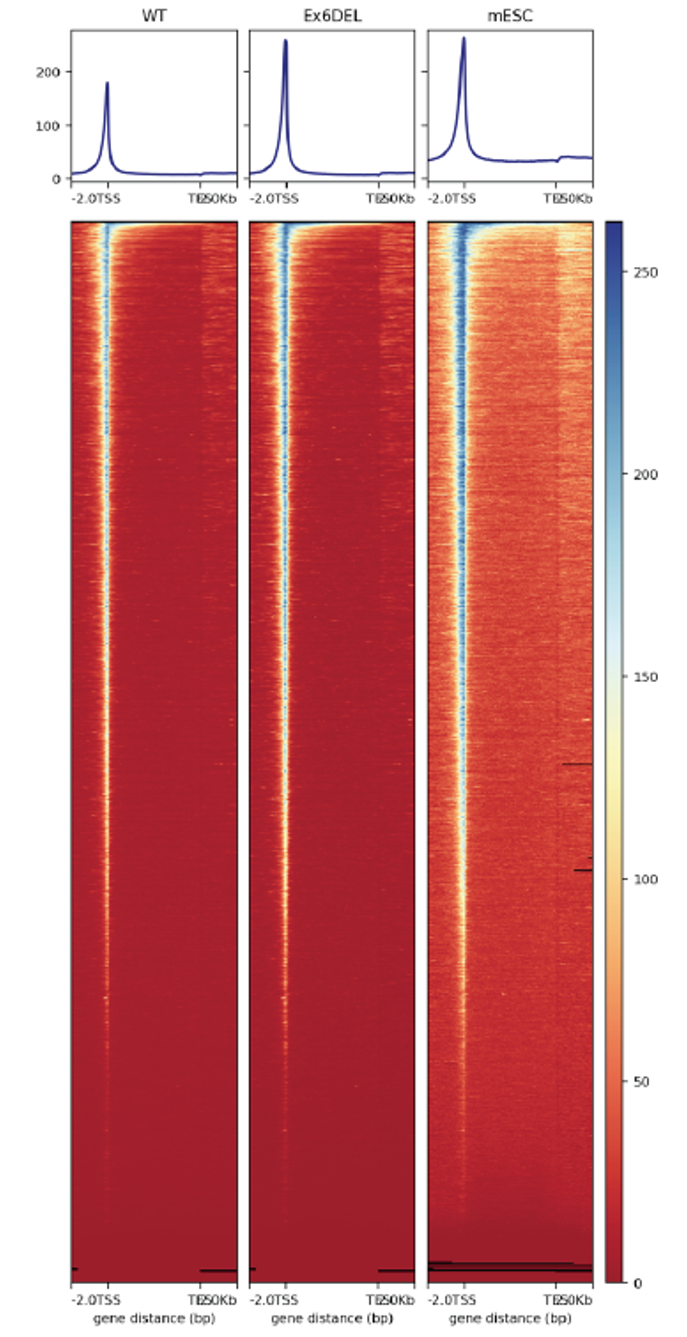


**Supplemental Figure 4. Genome wide chromatin accessibility at TSS.** Chromatin accessibility maps were generated by normalizing all genes to an identical size and then showing the chromatin for each gene around the TSS. Accessibility was increased for the Ex6DEL (middle panel) GNP cultures compared to WT (left panel) cultures and the level of open chromatin was more similar to that observed for mouse ESCs (right panel).


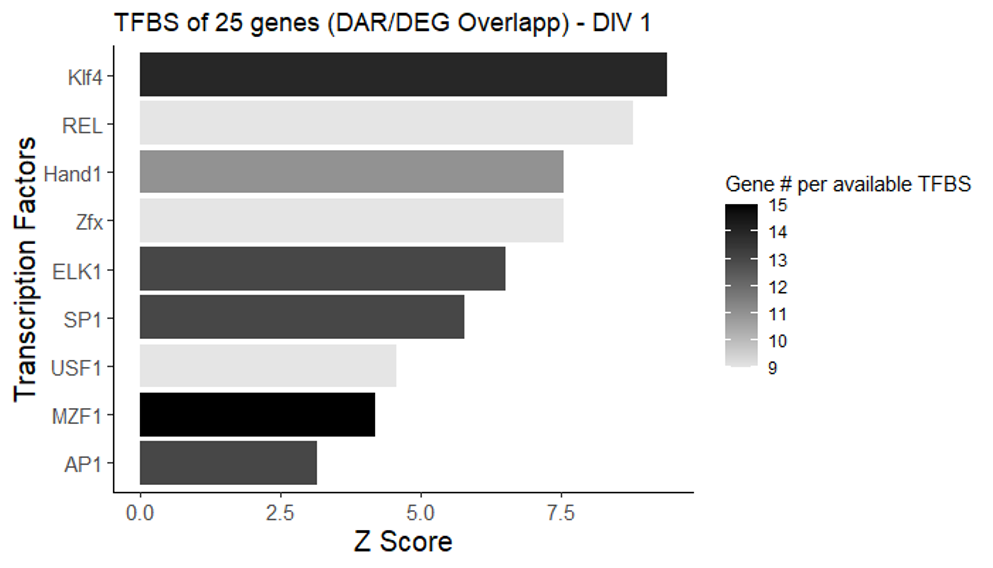


**Supplemental Figure 5. Transcription factor binding sites at DAR/DEG overlapping genes.** A total of 25 DEGs at 1 DIV had a corresponding DAR located within the promoter DAR. The graph plots the common transcription factor binding sites located within the promoter DAR of these DEGs.


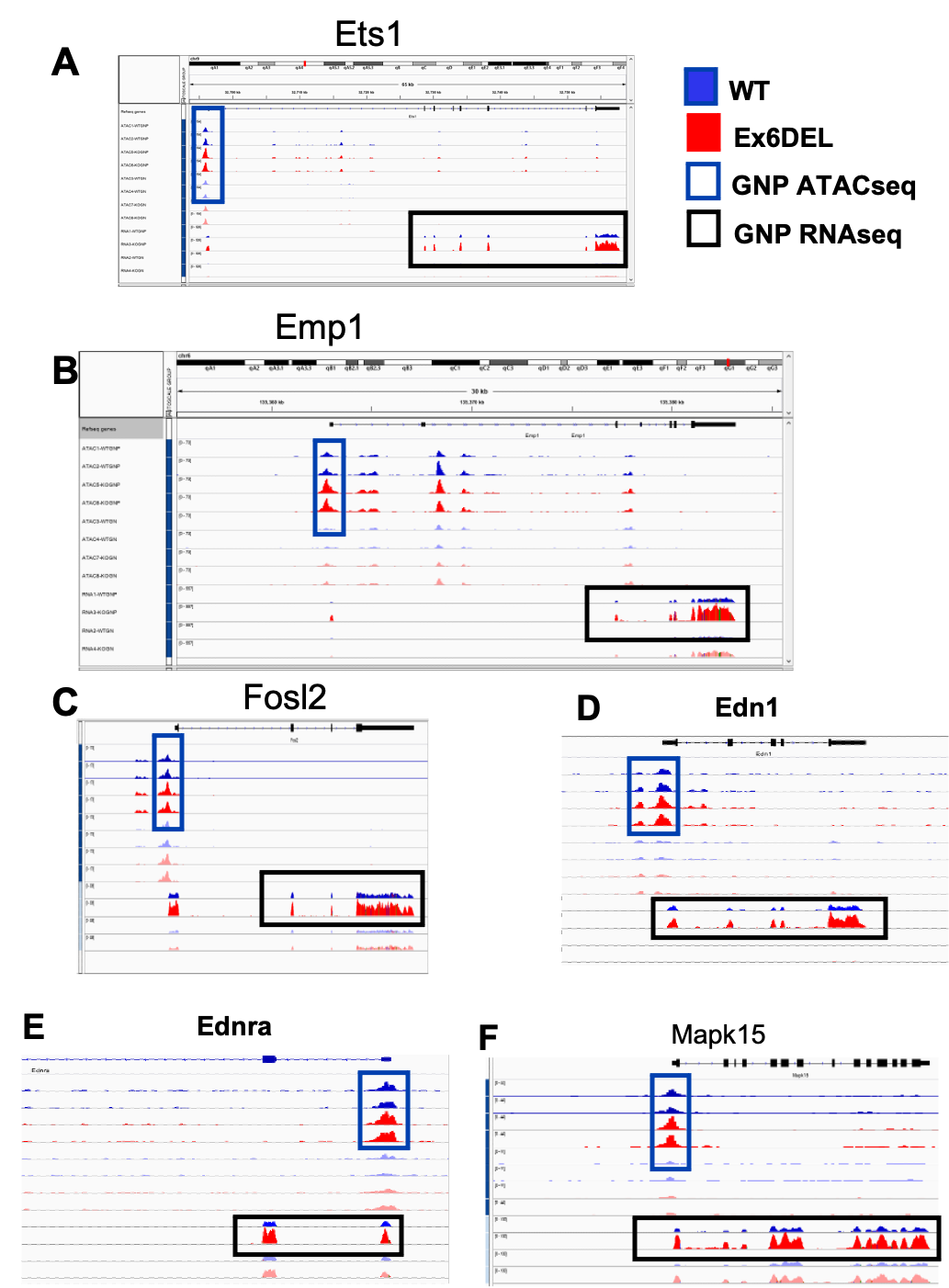
**Supplemental Figure 6.** **Genome browser tracks for selected DEGs**. ATACseq peaks and RNAseq reads are shown in IGV browser format for the *Ets1* **(A)**, *Emp1* **(B)***,* *Fosl2* **(C),** *Edn1* **(D)***, Ednra* **(E)***, and Mapk15* **(F)** genes. Peaks in blue correspond to data from WT cultures while red peaks correspond to Ex6DEL cultures. The 1 DIV GNP ATACseq tracks are shown in the blue box. The 1 DIV GNP RNAseq reads are shown within the black box. The remaining unboxed tracks correspond to ATACseq and RNAseq from the 3 DIV timepoints corresponding to GNs.


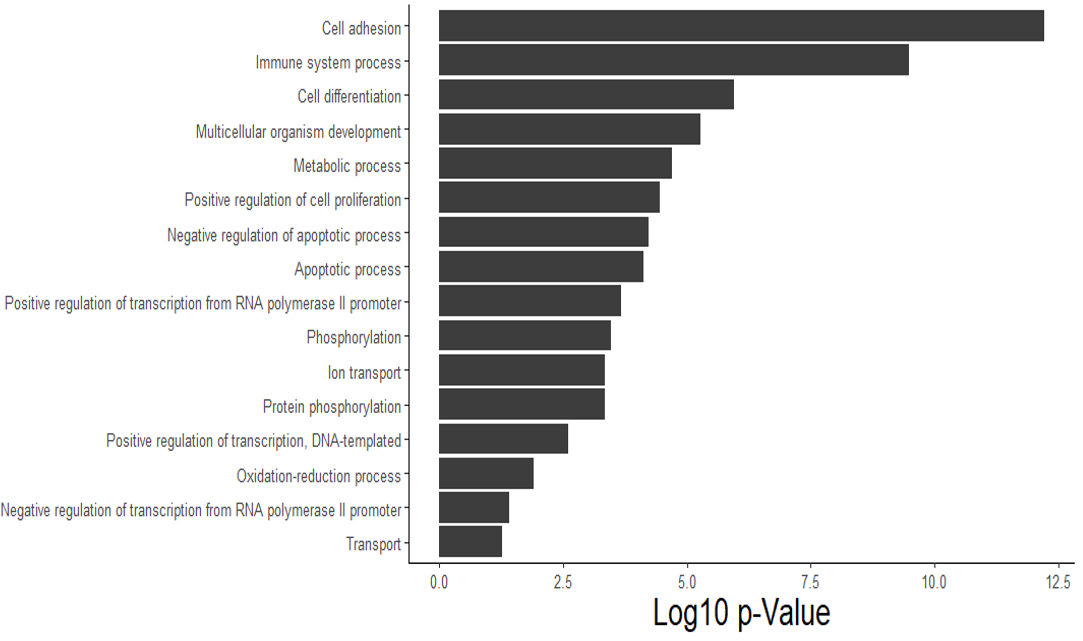


**Supplemental Figure 7. GO Term analysis for novel DEGs at 3 DIV timepoint.** Comparison of WT and Ex6DEL cultures at 3 DIV identified 2248 DEGs that were distinct from the Ex6DEL- and WT-specific DEGs changing between 1- and 3-DIV.
